# Application of Static Headspace GC-MS Method for Selective 1,4-Dioxane Detection in Food Additives

**DOI:** 10.3390/foods12173299

**Published:** 2023-09-02

**Authors:** Myung-Chan Kim, Su-Yeon Park, Seo-Yeon Kwon, Yu-Kyeong Kim, Yeong-In Kim, Yong-Soo Seo, Sueng-Mok Cho, Eui-Cheol Shin, Jin Hong Mok, Yang-Bong Lee

**Affiliations:** 1 Department of Food Science and Technology, Pukyong National University, Busan 48513, Republic of Korea; michael4626@naver.com (M.-C.K.); suzan@pukyong.ac.kr (S.-Y.P.); seoyeon529@pukyong.ac.kr (S.-Y.K.); 12kug18@gmail.com (Y.-K.K.); inkim@pukyong.ac.kr (Y.-I.K.); scho@pknu.ac.kr (S.-M.C.); jhmok1024@pknu.ac.kr (J.H.M.); 2 Cooperative Laboratory Center, Pukyong National University, Busan 48513, Republic of Korea; yongsoo7@pknu.ac.kr; 3 Department of Food Science/GreenBio Science, Gyeongsang National University, Jinju 52828, Republic of Korea; eshin@gnu.ac.kr

**Keywords:** polyethylene glycol, method validation, limit of detection, limit of quantification

## Abstract

Efficient detection methods must be developed for 1,4-dioxane due to its suspected status as a human carcinogen, which is highly mobile in food and environmental resources. In this regard, this experiment has been conducted to develop reliable and selective detection and measurement methods by using static headspace (SH) isolation, followed by gas chromatography–mass spectrometry (GC-MS). A new method was developed for determining the spiked 1,4-dioxane contents in a polyethylene glycol 600 (PEG 600). The optimal condition for SH-GC-MS was discussed. The representative ions of 1,4-dioxane and 1,4-dioxane-d8 in the SIM mode of MS are 88 and 96, respectively, and the peaks of the SIM mode were separated and confirmed. The linear range for the method covers 0.25 to 100 mg/L with a coefficient of determination (R^2^)  ≥  0.999. The method applicability was demonstrated by spike recovery across a variety of food additives (i.e., chlorine bitartrate, choline chloride, polysorbate 20 and 60, and PEG 1000). All spike recovery from the tested samples was in the range of 89.50–102.68% with a precision of 0.44–11.22%. These findings suggest a new analytical method for food safety inspection, and could be applicable for ensuring the safety of foods and environmental and public health on a broad scale.

## 1. Introduction

1,4-Dioxane is a contaminant of emerging concern that has been widely detected not only in groundwater but also in pre- and post-food manufacturing. For example, it can be formed by the acid-catalyzed dehydration of ethylene oxide, which can be found in the manufacturing step of ethoxylated food additives, such as choline chloride, chlorine bitartrate, polysorbate, and polyethylene glycol (PEG) [1,2]. Many agencies, safety protocols, and research reports have classified 1,4-dioxane as a substance that can cause liver damage and kidney failure, and its induced genotoxicity and carcinogenesis have been studied in numerous in vitro and in vivo experiments [3,4,5,6,7,8]. In addition, considering its boiling point (about 101 °C), it is not easily volatilized during food processing (ethoxylation process), and so would remain as is [9].

Due to these critical issues, the regulation of 1,4-dioxane utilization and management became strict through methods such as monitoring its significant changes with a unit of threshold limit value (TLV) by time-weighted average (TWA), or giving it an extremely low recommended exposure limit (REL), such as 1 ppm, and classifying it as immediately dangerous to life or health (IDLH), which it is at about 500 ppm [10]. Consequently, to detect such a low level of chemicals with high accuracy and precision, it has to be processed with an appropriate sample pretreatment step, preconcentration or calibration of trace analytes, isolation from a complex matrix, or separation from components interfering in employed detection. To date, a number of conventional methods have been applied for 1,4-dioxane detection and quantification successfully, including purge-trap gas chromatography–mass spectrometer (PT-GC-MS), direct aqueous injection, and GC-MS analysis of continuous liquid–liquid extraction (LLE). The U.S. EPA suggests the purge-trap technique and liquid–liquid extraction for 1,4-dioxane isolation from various samples, such as drinking water, shampoo, liquid soap, choline salt, polysorbate, and PEG [11,12,13,14,15]. In addition, purge-trap combined with gas chromatography–flame ionization detection (GC-FID) can be performed with small sample volumes and multiplexing capabilities for high-throughput processing [16]. However, most of these methods for the determination of the chemical compounds and toxins very often require a high cost, such as PT-GC-MS or -FID, and long periods for the preparation of samples for LLE step. To overcome this, in the last two decades, flow analytical methods with an efficient and convenient sample technique and the electrochemical technique for the rapid sensing of 1,4-dioxane have been developed. But faster methods for a preconcentration phase, which refers to the process of concentrating a sample so that trace components are not lost before analysis, are still needed for emergency response, as well as for public health and environmental protection.

The static headspace (SH) method is a simple and direct injection, which is also advantageous for the sampling and isolation of volatile compounds for the extraction step. Several studies on SH-GC-MS for cosmetic quality screenings for 1,4-dioxane have been reported and applied to their regulation claims [17,18,19]. However, there is limited information on the capability of SH-GC-MS to quantify 1,4-dioxane when it spikes in the food additive matrices. With a similar approach for facilitating the simple assay of a large number of sample matrices and efficient sample isolation, we applied a combination platform of a SH method and a gas chromatograph–mass spectrometer (GC-MS) as an analytical method to increase selectivity against 1,4-dioxane in food samples. Additionally, the representative ions of 1,4-dioxane and 1,4-dioxane-d8 in the SIM mode of MS are 88 and 96, respectively, and the accuracy of quantitative analysis can be improved by adopting the ratio of their peak areas [9,20]. To find out the optimal conditions for 1,4-dioxane isolation, the SH and validation of the suggested methods regarding the incorporation of additives into food matrices are described.

## 2. Materials and Methods

### 2.1. Chemicals, Standards, and Reference Materials

1,4-Dioxane (99.5%) was a product of Daejung Chemicals & Metals (Siheung-si, Republic of Korea), and 1,4-dioxane-d8 (2000 μg/mL in methanol), which was used as an internal standard (IS) material, was purchased from Sigma-Aldrich (Saint Louis, MO, USA). Choline bitartrate (98%), choline chloride (99%), polysorbate 60 (≥99.5%), and PEG 600 (≥99.5%), used as bases in the preparation of standard solutions, were products of Daejung Chemicals & Metals. Polysorbate 20 (99%) was purchased from Duksan reagents (Ansan-si, Republic of Korea), and PEG 1000 (≥99.5%) was purchased from Samchun Chemicals (Seoul, Republic of Korea).

For crimp-top headspace analysis, 20 mL vials (TLS-20ML-20-V1002), 0 mm PTFE/white silicone, 3 mm thick septa (TLS-20-SP3004), and 20 mm open-top aluminum crimp caps (10 mm hole) were purchased from Yuil Labtech (Namyangju-si, Korea). For comparisons, a screw-type head space with 20 mL vials (SL.Vi1173) and caps (SL.Vi1176) were purchased from SciLab Korea (Seoul, Republic of Korea).

### 2.2. Standard Solution

1,4-Dioxane standard solutions were prepared in the concentration range from 15 up to 6000 mg/L. With 3 g of PEG 600 sample for the standard sample, their final concentrations were in the range of 0.25–100 mg/L (50 μL of the standard solution was added).

For an IS, 1,4-dioxane-d8 standard solution was prepared at a concentration of 600 mg/L using distilled water in consideration of the regulated concentration (5.0 ppm for polysorbate in the Food Additive Code, 10 ppm for the others). The IS solution of 50 μL was added to 3 g of the PEG 600 used for the standard samples and its final concentration became 10 mg/L. The standard and IS were transferred into GC–MS vials with screw top caps and stored at 0–10 °C until use for further analysis.

### 2.3. Sample Preparation

**Figure 1:**
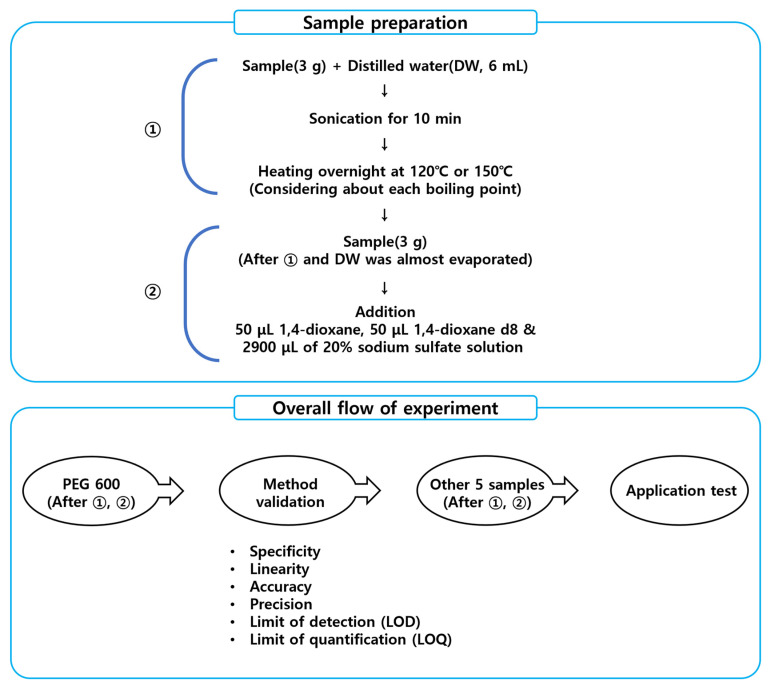
Schematic diagram of sample preparation and experimental procedures in SH-GC-MS for 1,4-dioxane quantification.

An explanation of the overall procedure of the experiment is shown in Figure 1.

To remove 1,4-dioxane present in the sample, when preparing a base of samples, 3 g each of choline bitartrate, choline chloride, polysorbate 20, polysorbate 60, PEG 600, and PEG 1000 was subdivided into a vial before use, and 6 mL of distilled water (DW) was added, and then ultrasonically dispersed for 10 min. For reference, the reason for adding water is to remove existing 1,4-dioxane by using the water-soluble property and it was judged based on whether it measured below the LOD by GC-MS. Then, considering the boiling point of each base, choline bitartrate was overnight at 120 °C and the rest at 150 °C to prepare samples.

**Figure 2:**
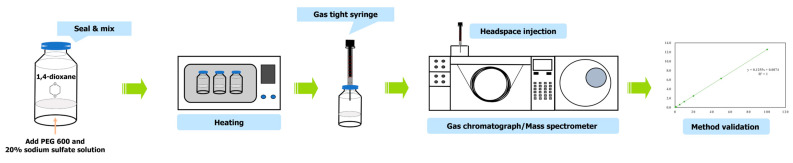
Schematic diagram for SH-GC-MS for 1,4-dioxane detection and measurement experiment.

Sample preparation was carried out with a little modification from ‘Guidelines for Analysis of Prohibited Ingredients in Cosmetics’ in Korea [17]. For the pre-treatment of PEG 600, which is a representative base for validation, 1–3 g of the sample was taken and put in 20 mL of screw-type and crimp-top type headspace vials, 2950 μL of 20% sodium sulfate solution and IS solution (1,4-dioxane-d8) was added to test solution. To prepare a sample for preparing a calibration curve and measuring recovery yield, 3 g of base was taken and put in a headspace vial to consider the effect of the matrix, 2900 μL of 20% sodium sulfate solution, 50 μL of IS solution (1,4-dioxane-d8) was added to make 0.25, 0.5, 1, 5, 10, 20, 50 and 100 mg/L concentrations, respectively. Then, a constant temperature water bath (NB-301, N-Biotek, Bucheon, Republic of Korea) was used for target substance extraction, and the experiment was performed at the constant temperature between 20–90 °C for 30 min by water bath method (Figure 2).

Establishment of optimal conditions for 1,4-dioxane analysis by the SH method was carried out based on the analysis method of the Guidelines for Analysis of Incompatible Components in Cosmetics [17]. For validation to establish the optimal conditions for 1,4-dioxane analysis by the headspace method, the amount of sample, capping type, salt addition type, water bath temperature, and injection volume for GC analysis were determined. For confirmation purposes, the accuracy and precision were estimated by two choline products, two polysorbate products, and one PEG product.

### 2.4. GC-MS Analysis

With the prepared samples described in a previous session, 100, 200, and 1000 μL headspace were manually injected using a 5 mL gastight syringe (Hamilton, model 1005 SL, Reno, NV, USA). A gas chromatograph (GC-2010 plus, Shimadzu Co., Kyoto, Japan) and mass spectrometry detector (GC-MS-QP2020, Shimadzu Co., Kyoto, Japan) were used for 1,4-dioxane isolation (and separation). DB-Wax capillary columns (Agilent Co., Santa Clara, CA, USA) with a size of 60 m × 0.32 mm and a film thickness of 0.5 μm were used. The flow rate was set to 1.5 mL/min. The oven temperature was maintained at 120 °C for 5 min, then raised to 140 °C at the heating rate of 10 °C/min and then heated to 200 °C at the heating rate of 30 °C/min, and maintained for 3 min. The inlet temperature was 180 °C, and the injection mode was 10:1 split mode. The interface temperature of the mass spectrometry detector (MSD) was 220 °C, the ion source temperature was 230 °C, and the electron impact energy was 70 eV. Selective ion monitoring (SIM) was applied 88 *m*/*z* for 1,4-dioxane and 96 *m*/*z* for 1,4-dioxane-d8.

### 2.5. Method Validation

The analytical method for 1,4-dioxane measurement in food additives was performed as suggested in the guidelines presented in the ‘Guideline Guidelines for Validation of Test Methods for Pharmaceuticals’, etc. [21]. It includes specificity, linearity, intra- and inter-day accuracy, intra- and inter-day precision, the limit of detection (LOD), and the limit of quantification (LOQ). For the method detection limit (MDL), all procedures were followed as described in ‘Definition and Procedure for the Determination of the Method Detection Limit, Revision 2’ from EPA [22]. The specificity test was followed. For a standard sample of PEG 600 for GC-MS, the chromatogram of the standard sample was obtained with the selected concentration of 1,4-dioxane (50 mg/L) and IS solution (1,4-dioxane-d8, 10 mg/L). For the linearity test, the concentrations of 1,4-dioxane were set to 0.25, 0.5, 1, 5, 10, 20, 50 and 100 mg/L. The correlation coefficient, y-intercept and slope were obtained from the regression line, and the linearity was confirmed by the correlation coefficient (R^2^) value. The calibration curve with an R^2^ value of 0.999 or more was selected and used. LOD and LOQ were calculated based on the signal-to-noise ratio (S/N). It is stated that 10 times for the detection limit and 3 times for the quantification limit are appropriate, so after repeating experiments (*n* = 5) for the standard solutions, the detection limit and quantification are expressed in the following formula. The determination of LOD and LOQ was discussed later. MDL was calculated by the formula of (t_(df,0.99)_* standard deviation (SD)) by repeating experiments (*n* = 7) by selecting a concentration with a S/N value of 10–20 [22]. Accuracy and precision were tested repeatedly (*n* = 3) by preparing the standard solutions of three concentrations above the quantification limit, and by measuring intra- and intra-day changes. The recovery yield and relative standard deviation (RSD) were calculated and evaluated.

### 2.6. Statistical Analysis

For statistical data analysis, a one-way ANOVA and a LSD multiple comparison with a *p*-value of 0.05 were conducted using the R program (R-4.3.1).

## 3. Results and Discussion

### 3.1. Establishment of Optimized Conditions for 1,4-Dioxane Detection and Quantification in Polyethylene Glycol 600 (PEG 600)

For the accurate quantification of 1,4-dioxane, firstly, the experimental settings for increase in the extraction yield of 1,4-dioxane in the SH have been set as change the sample weights (Table 1). With such a small sample weight (1 g, in the present study) of 1,4-dioxane, there were detection failures, which would be significantly associated with false-negative results. On the other hand, in the case of tests with a higher sample weight of 3 g PEG 600, a similar peak area between 1,4-dioxane and 1,4-dioxane-d8, and more robust reproducibility were found.

In the tests with different capping types (Table 2), there was no significant difference in the peak area ratio results when it was tested with the crimp-top type and with the screw type; however, the obtained peak areas of 1,4-dioxane and 1,4-dioxane-d8 were higher when it tested with the crimp-top type. With consideration to avoid the failure from gas leakage and to improve their detection and quantification, the crime-top type was selected—it has an advantage in the trapping of volatile compounds, while they become pressurized with heating, and it was seen by their higher values and stability.

To test the results’ dependence on the salt contents, 1,4-dioxane was spiked into the various phases of food additives with solid and liquid phases of sodium sulfate (Table 3). From the peak area ratio of 1,4-dioxane and 1,4-dioxane-d8, most of the results by addition of 0.3 g anhydrous sodium sulfate solid phase presented larger SD (up to 30.4% RSD), except for the results from bitartrate (0.8% RSD). In the case of the addition of 20% sodium sulfate solution, most of RSD results were reduced, and all RSD results were in the range of 0.9–16.9%. Therefore, with respect to reduced RSD in averaged results (35.3% from anhydrous sodium sulfate solid phase < 10.3% from 20% sodium sulfate solution), the addition of 20% sodium sulfate solution was more reasonable for further analysis.

With tests under different temperatures (Table 4), the peak area ratio of 1,4-dioxane and 1,4-dioxane-d8 was varied and the highest value was found when it was tested at 50 °C. In the range of 20–50 °C, which is a relatively low-temperature setting in this study, the peak area ratio presented incremental trends. However, at higher temperatures of 70 and 90 °C, there was a significant reduction in values—it may be derived from induced gas leakage or turbulence by applied pressure and heat. To minimize their systematic instability, 50 °C was determined as the optimal temperature.

In the selection of GC injection volume (Table 5), the results of peak area ratios in higher injection volumes (200 and 1000 μL) presented significantly higher than those of the lowest injection volume (100 μL) in this study. Additionally, with no significant difference in the results from 200 and 1000 μL injection and with consideration of the risk of sample contamination and efficiency, 200 μL injection was determined as the optimal injection volume.

### 3.2. Method Validation

For the validation of optimized experimental conditions from the previous section, specificity, linearity, accuracy, precision, LOD, and LOQ were evaluated with different concentrations of 1,4-dioxane in various types of food additives.

**Figure 3:**
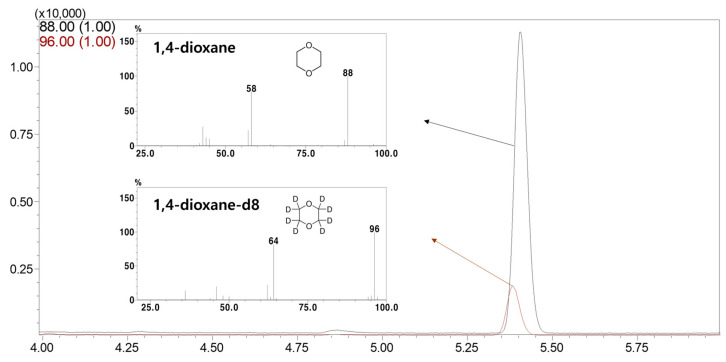
Chromatograms for standard solutions of 1,4-dioxane (50 mg/L) and 1,4-dioxane-d8 (10 mg/L). Represented selective ions of 1,4-dioxane and 1,4-dioxane-d8 are 88 and 96 *m*/*z*, respectively.

The specificity of 1,4-dioxane and 1,4-dioxane-d8 was confirmed (Figure 3), and each retention time was matched at 5.409 sec and 5.391 sec, respectively. The peak of the SIM mode component could be separated and confirmed.

**Figure 4:**
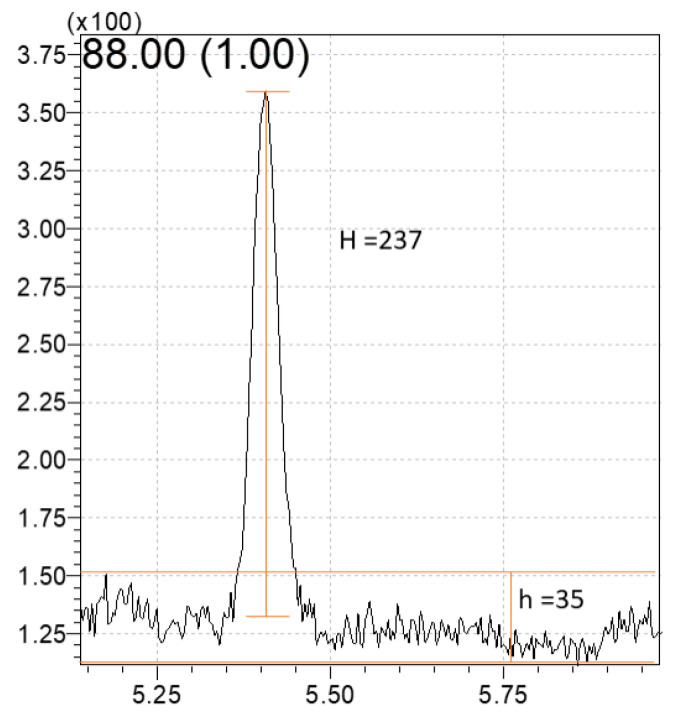
Chromatogram for calculating the S/N from the GC-MS signal chart (0.5 ppm 1,4-dioxane, S/N = 2H/h = 474/35 = 13.54). LOD = Standard concentration × 3/(S/N) = 0.5 × 3/13.57 = 0.11 ± 0.002 mg/L. LOQ = Standard concentration × 10/(S/N) = 0.5 × 10/13.57 = 0.37 ± 0.01 mg/L.

To determine the linearity, 1,4-dioxane levels were selected in the 1,4-dioxane concentrations of 0.25, 0.5, 1, 5, 10, 20, 50 and 100 mg/L. The linearity was based on the determination coefficients, which were higher than 0.9999 (Table 6), and their precision (RSD%) is confirmed between 0.71% to 16.40%. LOD and LOQ were calculated using a peak area ratio of 1,4-dioxane and 1,4-dioxane-d8 and the S/N for 0.5 mg/L concentration. Figure 4 presents a chromatogram for calculating S/N in the signal chart and the S/N information is shown in Table 7. Using these data, LOD and LOQ were evaluated based on the following equations (Figure 4). LOD and LOQ were 0.11 ± 0.002 mg/L and 0.37 ± 0.01 mg/L, respectively. These results were similar to data from Wang’s study [23], while lower than 0.2 μg/g LOD and 0.5 μg/g LOQ reported from Zhou’s study [9].

Since the LOD and LOQ are stated to be appropriate 1:10 (equivalent to 0.10) and 1:3 (equivalent to 0.33), respectively [9], both of LOD and LOQ of 1,4-dioxane, obtained in our study seemed to be reasonable. In the MDL calculation, as shown in Table 8, as described in EPA’s guideline, the S/N value from 1,4-dioxane concentration of 0.5 mg/L was selected and the final value was determined to 0.129 mg/L.

For the intra-day accuracy and precision of this SH-GC-MS method (Table 9), all recovery yields of different 1,4-dioxane concentrations were higher than 95.8 (up to 99.7%) with less than RSDs of 2.8%. In a similar way, the inter-day accuracy and precision test were estimated (Table 10). The inter-day accuracy was in the range of 96.8 to 101.0% with small RSDs (less than 2.0%). These intra- and inter-day results presented the reproducibility of the suggested SH-GC-MS for 1,4-dioxane detection and quantification.

For the applicability of SH-GC-MS for food additives, five standard samples were selected as base materials, and the results are shown in Table 11. In terms of recovery yield, it was confirmed that the range of recovery yield was 94.1 to 104.1% for choline bitartrate, 92.3 to 99.6% for choline chloride, 93.6 to 109.7% for polysorbate 20, 78.0 to 96.2% for polysorbate 60, and 92.0 to 103.1% for PEG 1000. All RSDs were in the range of 0.4–11.2%, and they met the suggested conditions (should be less than 20%) described in the ‘Food Additives Test Method of Guidelines for Standard Procedures for Food Test Methods’ by the Ministry of Food and Drug Safety, with respect to the verification factors and reference range. Therefore, this SH-GC-MS-based analytical method presented sufficient reliability and repeatability for 1,4-dioxane detection and quantification in food samples.

## 4. Conclusions

The analytical method of 1,4-dioxane in food additives referred to ‘Guidelines for Analysis of Prohibited Ingredients in Cosmetics’ [17]. The optimal pretreatment conditions were a 3 g sample amount, a crimp-top capping type, a salt addition type of 20% sodium sulfate solution, a 50 °C isolation temperature, and a 200 μL GC injection. The values obtained through method validation are 0.999 for R^2^, 0.11 mg/L for LOD, 0.36 mg/L for LOQ. The intra-day accuracy and precision are between 95.8–99.7% and 1.1–2.8%, respectively. The inter-day accuracy and precision are 96.8–101.0% and 0.6–2.0%, respectively. The applicability of five different base types is tested, with an accuracy of 89.5–102.7% and precision of 0.4–11.2%. The method verification and applicability results met the verification factors and standards of the food additive inspection method of the Ministry of Food and Drug Safety’s standard procedure guidelines. This study established an optimum experimental condition of static headspace isolation for 1,4-dioxane measurement, and the results are believed to be easier to analyze 1,4-dioxane remaining in food additives by using the static headspace method.

## Figures and Tables

**Table 1:** Summary of optimization of sample weight of PEG 600 for SH-GC-MS.

Concentration (mg/L)	Rep.	Sample Weight (g)					
		1.0			3.0		
		Peak Area		Ratio	Peak Area		Ratio
		1,4-Dioxane-d8	1,4-Dioxane		1,4-Dioxane-d8	1,4-Dioxane	
10	1	6205	9638	1.553	191,005	199,685	1.045
	2	nd	nd	-	216,076	227,540	1.053
	3	10,698	16,289	1.523	210,731	220,572	1.047
	4	nd	nd	-	295,323	302,288	1.024
	5	34,880	53,249	1.527	192,687	204,178	1.060
Average ± SD				1.53 ± 0.02 ^a^			1.05 ± 0.01 ^b^

Data were analyzed by ANOVA and the multiple comparisons of LSD. In samples 1 g and 3 g, the different superscripts in ratios mean significantly different at *p* < 0.05.

**Table 2:** Summary of cap type for optimized setting for SH-GC-MS.

Capping Type	Average ± SD (Ratio of 1,4-Dioxane/1,4-Dioxane-d8)
Crimp-top Screw	1.23 ± 0.02 ^a^
	1.21 ± 0.03 ^a^

Data were analyzed by ANOVA and the multiple comparisons of LSD. In the capping type of crimp-top and screw for sample vials, the same superscripts in average values mean significantly the same at *p* < 0.05.

**Table 3:** Summary of peak area information by the addition of salt in PEG 600 for optimized settings for SH-GC-MS.

Base (Phase)	Average Values of Peak Area Ratio of 1,4-Dioxane/1,4-Dioxane-d8	
	Anhydrous Sodium Sulfate Solid Phase	20% Sodium Sulfate Solution
PEG 600 (VL)	2.01 ± 0.11 ^b^	2.18 ± 0.02 ^b^
Bitartrate (S)	1.30 ± 0.01 ^b^	1.86 ± 0.08 ^b^
Polysorbate 20 (VL)	5.39 ± 1.64 ^a^	7.47 ± 1.26 ^a^
Polysorbate 60 (VL)	2.73 ± 0.37 ^b^	1.81 ± 0.04 ^b^
Total average ± SD	2.01 ± 0.71 ^A^	1.95 ± 0.20 ^A^

In the column, the different superscripts in average values mean significantly different at *p* < 0.05. In the case of the total average from bases, the same superscripts in a row mean no significant difference at *p >* 0.05. S—solid; VL—viscous liquid.

**Table 4:** Summary of the average peak area ratio of 1,4-dioxane and 1,4-dioxane-d8 at different isolation temperatures in PEG 600 by SH-GC-MS.

Temperature (°C)	Average Values of Peak Area Ratio of 1,4-Dioxane/1,4-Dioxane-d8
20	3.59 ± 0.21 ^c^
30	3.89 ± 0.04 ^bc^
40	3.97 ± 0.14 ^b^
50	4.98 ± 0.01 ^a^
70	4.18 ± 0.14 ^b^
90	2.18 ± 0.02 ^f^

Data were duplicated and analyzed by using R for ANOVA and the multiple comparisons of LSD. In isolation temperatures of 20, 30, 40, 50, 70, and 90 °C, the different superscripts in average values mean significantly different at *p* < 0.05.

**Table 5:** Summary of the average peak area ratio of 1,4-dioxane and 1,4-dioxane-d8 at different injection volumes in PEG 600 for SH-GC-MS.

Injection Volume (μL)	Average Values of Peak Area Ratio of 1,4-Dioxane/1,4-Dioxane-d8
100	1.41 ± 0.01 ^b^
200	1.56 ± 0.03 ^a^
1000	1.53 ± 0.05 ^a^

Data were duplicated and analyzed by ANOVA and the multiple comparisons of LSD. In injection volumes 100 μL, 200 μL, and 1000 μL, the different superscripts in average values mean significantly different at *p* < 0.05.

**Table 6:** Results of linearity of 1,4-dioxane concentration established by the static headspace analysis in PEG 600.

1,4-Dioxane Concentration (mg/L)	Replication (*n* = 3)			Average ± SD	RSD (%)
	*n* = 1	*n* = 2	*n* = 3		
0.25	0.054	0.047	0.065	0.06 ± 0.01	16.40
0.5	0.093	0.084	0.099	0.09 ± 0.01	8.21
1	0.15	0.151	0.127	0.14 ± 0.01	9.52
5	0.633	0.631	0.602	0.62 ± 0.02	2.79
10	1.287	1.295	1.266	1.28 ± 0.01	1.17
20	2.505	2.531	2.497	2.51 ± 0.02	0.71
50	6.393	6.405	6.279	6.36 ± 0.07	1.09
100	12.742	12.747	12.571	12.69 ± 0.10	0.79
Slope	0.127	0.127	0.126	0.127 ± 0.001	-
Intercept	0.011	0.012	0.007	0.010 ± 0.003	
R^2^	1	1	1	-	

**Table 7:** Results of signal-to-noise ratio (S/N) at the standard concentration of 0.5 mg/L.

No.	Ratio of 1,4-Dioxane Peak Area/1,4-Dioxane-d8 Peak Area	S/N
1	0.093	13.54
2	0.089	13.68
3	0.099	13.28
4	0.090	13.41
5	0.098	13.95
Average ± SD	0.094 ± 0.005	13.57 ± 0.26
RSD (%)	4.85	1.90

**Table 8:** Results of detection contents and recovery yields were obtained by measuring 1,4-dioxane of 0.5 mg/L for MDL determination in PEG 600.

1,4-Dioxane			
Spike Concentration	0.5 mg/L		
Experimental Run	Ratio *	Detection Amount (mg/L)	Recovery (%)
1	0.093	0.633	127
2	0.089	0.601	120
3	0.099	0.680	136
4	0.090	0.609	122
5	0.098	0.672	134
6	0.088	0.593	119
7	0.085	0.570	114
Average ± SD	-	0.623 ± 0.041	124.51 ± 8.19
Degree of freedom (=*n* − 1)	-	6	-
t (*n* − 1, 1 − α = 0.99)	-	3.143	-
MDLs (=t × SD)	-	0.129(=3.143 × 0.041)	-

* Ratio means 1,4-dioxane peak area/1,4-dioxane-d8 peak area.

**Table 9:** Intra-day results of recovery yield and RSD of 1,4-dioxane in PEG 600 by SH-GC-MS.

1,4-Dioxane Concentration (mg/L)	Intra-Day	Detection Amount (mg/L)	Recovery Yield (%)	Average ± SD	RSD (%)
5	Day 0	4.874	97.5	95.8 ± 2.7	2.8
		4.859	97.2		
		4.631	92.6		
10	Day 0	10.003	100.0	99.7 ± 1.2	1.2
		10.068	100.7		
		9.843	98.4		
50	Day 0	50.085	100.2	99.6 ± 1.1	1.1
		50.175	100.3		
		49.185	98.4		

**Table 10:** Inter-day results of recovery yield and RSD of 1,4-dioxane in PEG 600 by SH-GC-MS.

1,4-Dioxane Concentration (mg/L)	Inter-Day	Detection Amount (mg/L)	Recovery Yield (%)	Average ± SD	RSD (%)
5	Day 1	4.895	97.9	96.8 ± 1.3	1.3
		4.859	97.2		
		4.769	95.4		
10	Day 2	10.069	100.7	101.0 ± 0.6	0.6
		10.068	100.7		
		10.172	101.7		
50	Day 3	48.865	97.7	98.2 ± 2.0	2.0
		50.175	100.3		
		48.253	96.5		

**Table 11:** Results of detection amounts and recovery yields of 1,4-dioxane in 5 different types of food additives.

1,4-Dioxane Concentration (mg/L)	Choline Bitartrate			Choline Chloride			Polysorbate 20			Polysorbate 60			PEG 1000		
	Detection Amount (mg/L)	Recovery Yield (%)	RSD (%)	Detection Amount (mg/L)	Recovery Yield (%)	RSD (%)	Detection Amount (mg/L)	Recovery Yield (%)	RSD (%)	Detection Amount (mg/L)	Recovery Yield (%)	RSD (%)	Detection Amount (mg/L)	Recovery Yield (%)	RSD (%)
5	4.860	97.2	1.64	4.616	92.3	3.22	4.922	98.4	1.22	4.647	92.9	1.07	4.599	92.0	3.16
	4.814	96.3		4.737	94.7		5.001	100.0		4.634	92.7		4.773	95.5	
	4.706	94.1		4.920	98.4		4.883	97.7		4.556	91.1		4.899	98.0	
10	9.411	94.1	0.72	9.641	96.4	1.01	9.365	93.6	2.90	8.545	85.4	4.99	9.971	99.7	2.95
	9.431	94.3		9.462	94.6		9.924	99.2		9.442	94.4		9.718	97.2	
	9.537	95.4		9.492	94.9		9.679	96.8		9.050	90.5		10.306	103.1	
50	52.037	104.1	3.19	49.440	98.9	0.44	54.841	109.7	6.39	38.981	78.0	11.22	49.424	98.8	1.08
	50.815	101.6		49.819	99.6		50.848	101.7		47.160	94.3		48.432	96.9	
	48.843	97.7		49.451	98.9		48.340	96.7		48.114	96.2		49.244	98.5	

## Data Availability

The data presented in this study are available on request from the corresponding author.
